# Supplementary material for: Host Cell Redox Alterations Promote Latent HIV-1 Reactivation through Atypical Transcription Factor Cooperativity
Source: Viruses. 2022 Oct 18;14(10):2288. doi: 10.3390/v14102288 (PMC9612055; doi:10.3390/v14102288)
Supplement: Supplementary file 1 [file viruses-14-02288-s001.zip › Supplemental Material.pdf]

Supplemental Material

Supplemental figures

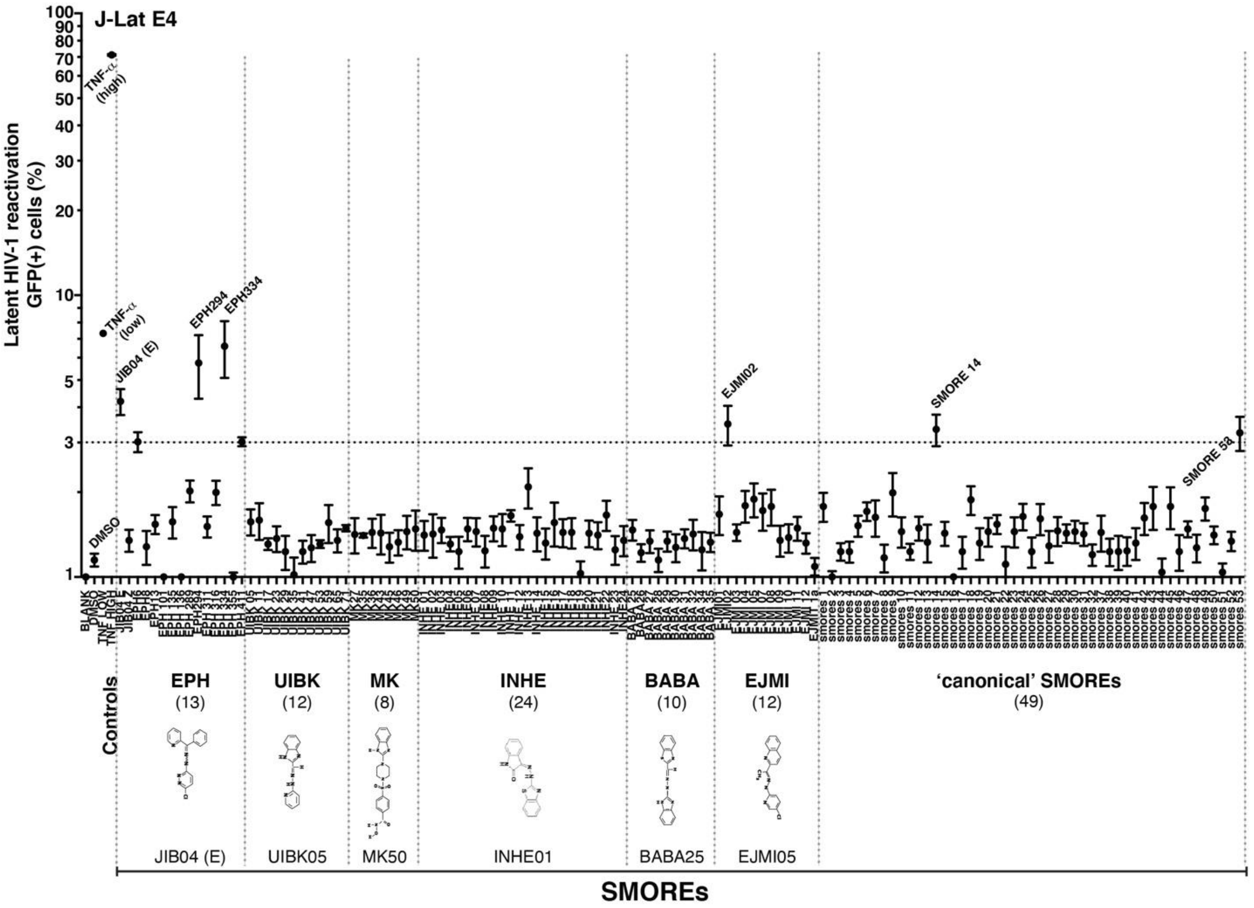

Supplementary Fig. S1. SMOREs screen in J-Lat E4 cell model of latency

J-Lat E4 cells were treated as indicated in the text and latent HIV-1 reactivation measured by flow cytometry to assess levels of GFP expression. The number of compounds per group is indicated in brackets and representative chemical structures of one compound per group are shown. The dashed line differentiates compounds that activate more than 3-fold over control samples (DMSO) compared to those that do not activate or activate less than 3-fold. TNF-α high (10 ng/mL) or low (1 ng/mL) concentrations were used as positive controls to gauge the dynamic range of the response. The percentage of GFP positive cells from three independent runs is indicated (mean ±SEM; n = 3).

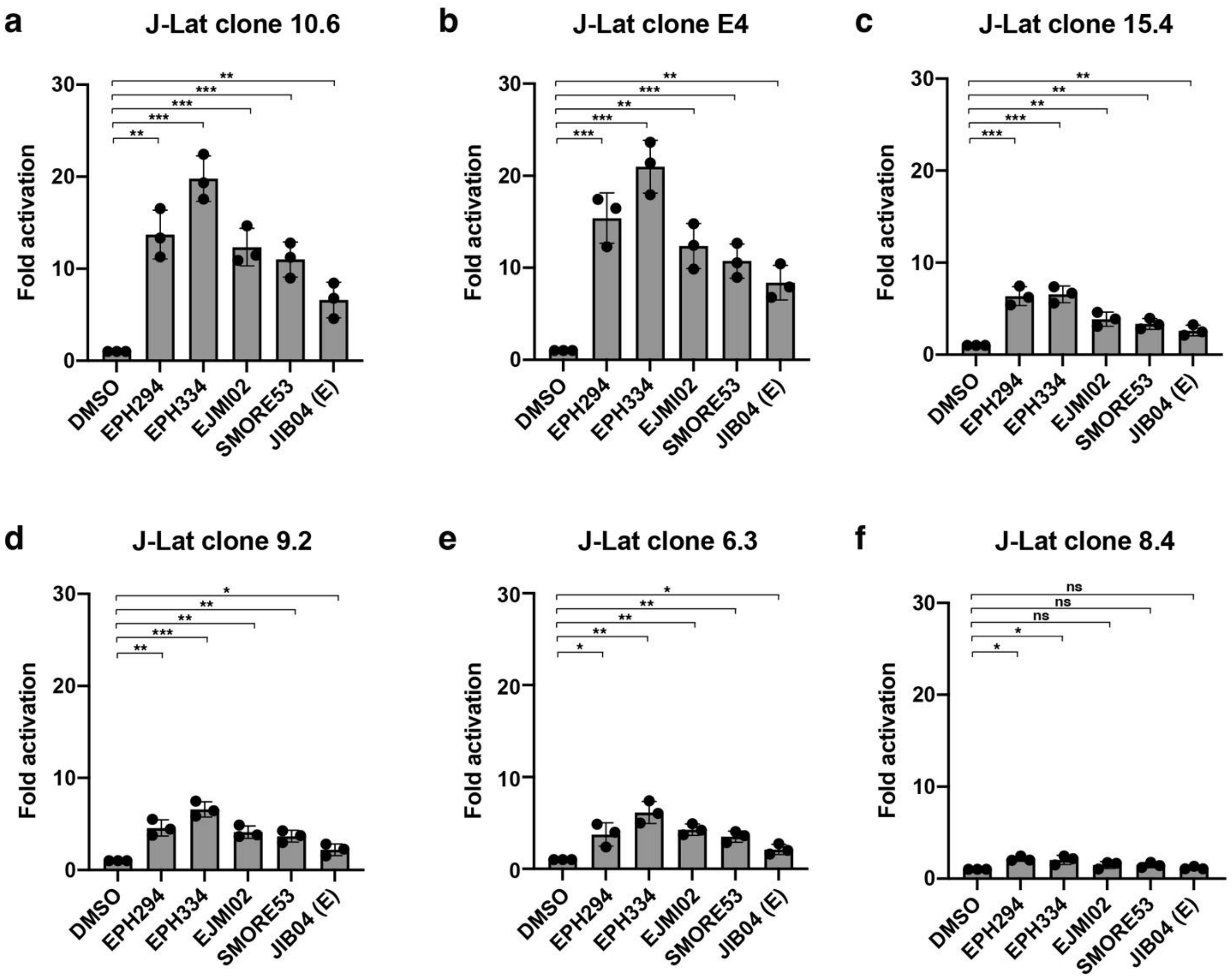

Supplementary Fig. S2. SMOREs reactivate latent HIV-1 from several euchromatin cell models of latency

a-f) Various J-Lat cell models were treated with the indicated SMOREs (1 μM) for 24 hrs and RNA isolated for RT-qPCR for quantitation of HIV-1 RNAs (VQA amplicon) normalized to *RPL19* (mean ± SEM; n = 3). P-values (*P < 0.05, **P < 0.01, ***P < 0.001) were determined by unpaired Student's t test. P < 0.05 was considered statistically significant.

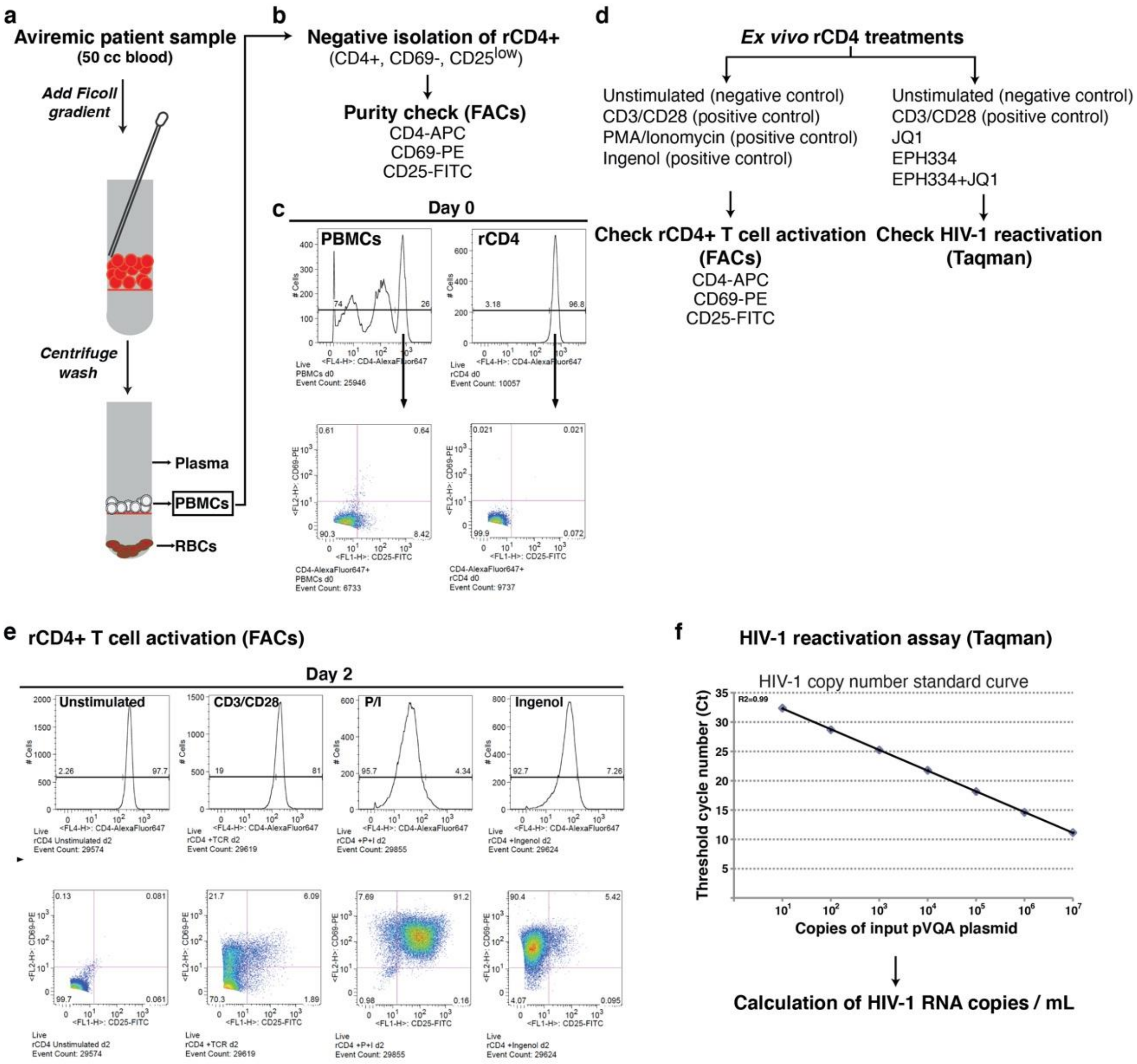

Supplementary Fig. S3. Purification and *ex vivo* treatment of patient-derived resting CD4^{+} T cells for latent HIV-1 reactivation assays

a-b) Schemes depicting the steps of resting (rCD4^{+}) T cell isolation from aviremic patient samples and markers used for purity check.

c) Flow cytometry measurement of CD4-APC (top), and CD69-PE and CD25-FITC (bottom) in PBMCs and rCD4^{+} T cells.

d) Scheme depicting the *ex vivo* treatments performed on the rCD4^{+} T cells.

e) Flow cytometry measurement of rCD4^{+} T cell activation *ex vivo* 48 hrs post-stimulation.

f) HIV-1 copy number standard curve obtained through Taqman assays to measure HIV-1 RNA copies / mL of supernatant in patient samples of panel (e).

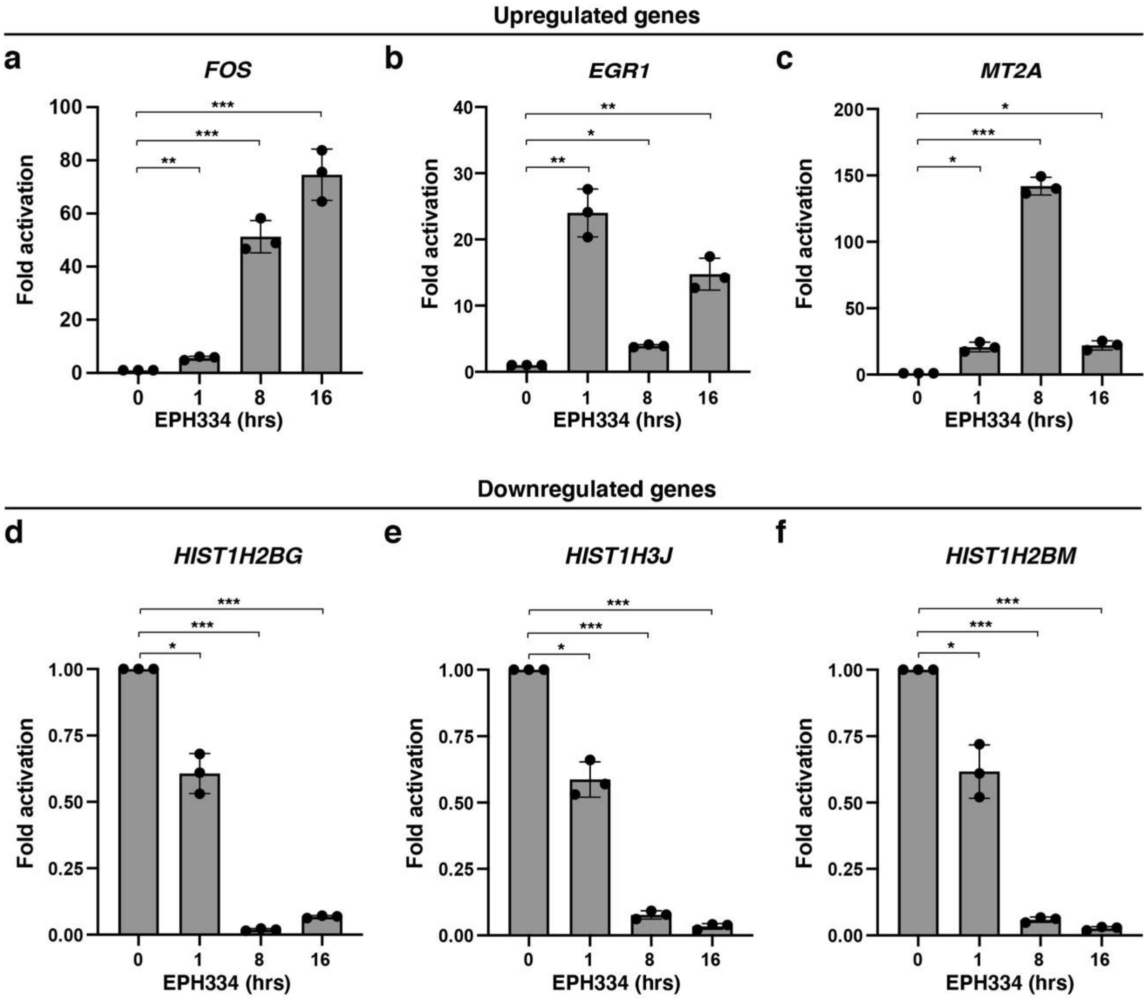

Supplementary Fig. S4. RNA-seq validation using RT-qPCR assays in J-Lat 10.6 cell model of latency

a-c) Quantitation of three upregulated genes from the RNA-seq dataset of Fig. 3 in response to a temporal EPH334 (2.5 μM) treatment and normalized to *U6* by RT-qPCR (mean ± SEM; n = 3). P-values (*$P < 0.05$, **$P < 0.01$, ***$P < 0.001$) were determined by unpaired Student's t test. $P < 0.05$ was considered statistically significant.

d-f) Quantitation of three downregulated genes from the RNA-seq dataset of Fig. 3 in response to a temporal EPH334 (2.5 μM) treatment and normalized to *U6* by RT-qPCR (mean ± SEM; n = 3). P-values (*$P < 0.05$, **$P < 0.01$, ***$P < 0.001$) were determined by unpaired Student's t test. $P < 0.05$ was considered statistically significant.

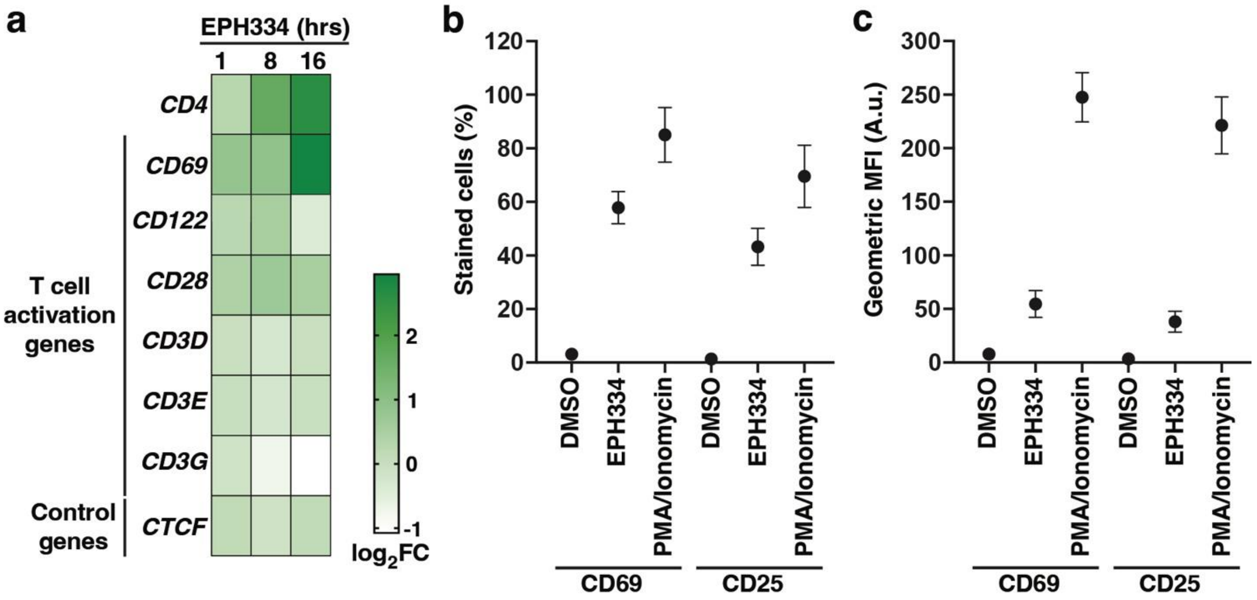

Supplementary Fig. S5. Expression of T cell activation genes in $CD4^+$ T immortalized and primary cells in response to EPH334 treatment

a) Heatmap showing the $\log_2$FC expression of the indicated genes in the J-Lat 10.6 cell model of latency treated with EPH334 (2.5 μM) for the indicated time points from the RNA-seq dataset of Fig. 3. CD25 was induced with EPH334; however, it was undetectable in untreated cells (0 read counts), thus hindering calculation of fold induction upon EPH334 treatment.

b) Flow cytometry measurement of the percentage (%) of stained cells for the indicated cell surface markers in resting memory $CD4^+$ T cells treated with DMSO (vehicle control), EPH334 (2.5 μM) or PMA/Ionomycin (10 ng/mL PMA and 0.1 μM Ionomycin) for 24 hrs. The percentage of positive cells from three independent runs from one representative donor is indicated (mean ± SEM; n = 3). Staining with isotype controls antibodies was done as negative control and revealed no staining.

c) Flow cytometry measurement of the geometric mean fluorescence intensity (MFI) of the samples of panel b). A.u. denotes arbitrary units.

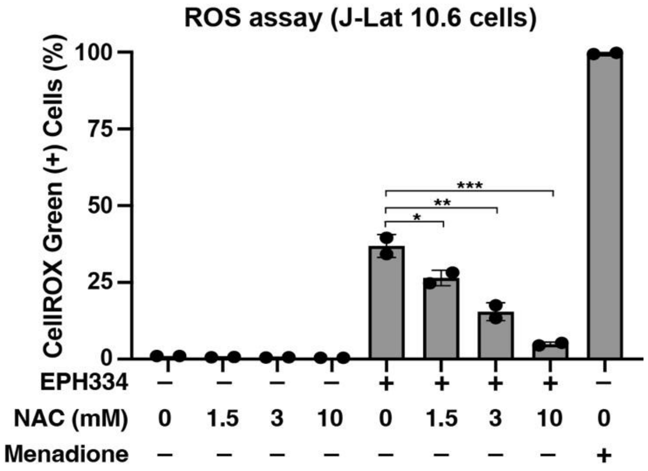

Supplementary Fig. S6. Dose-dependent N-Acetylcysteine–mediated EPH334-induced ROS inhibition in J-Lat 10.6 cell model of latency

Measurement of oxidative stress in J-Lat 10.6 cells pre-treated for 2 hrs with increasing concentrations of N-Acetylcysteine (NAC) and then treated with vehicle (DMSO) or EPH334 (2.5 μM) for 1 hr as judged based on the percentage of CellROX Green Dye positive cells determined by flow cytometry (n = 2). Excess Menadione (100 μM) was used as a positive ROS inducer control. P-values (*P < 0.05, **P < 0.01, ***P < 0.001, ns = not significant) were determined by unpaired Student's t test. P < 0.05 was considered statistically significant.

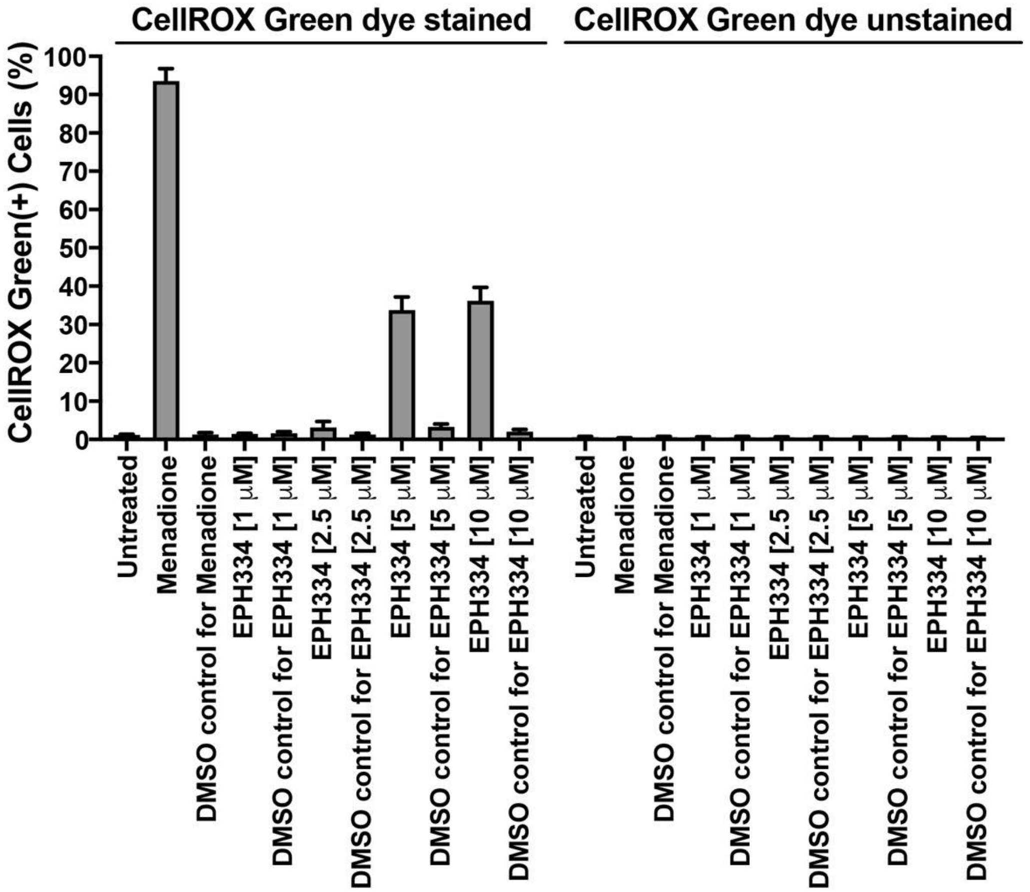

Supplementary Fig. S7. Measurement of oxidative stress derives from staining cells with the CellROX Green dye and not from GFP encoded from the provirus integrated into J-Lat 10.6 cells

Measurement of oxidative stress in J-Lat 10.6 cells treated with vehicle (DMSO), Menadione (100 μM) or EPH334 (1, 2.5, 5 and 10 μM) for 1 hr and then stained or not with CellROX Green Dye followed by flow cytometry analysis (n = 3). Excess Menadione (100 μM) was used as a positive ROS inducer control.

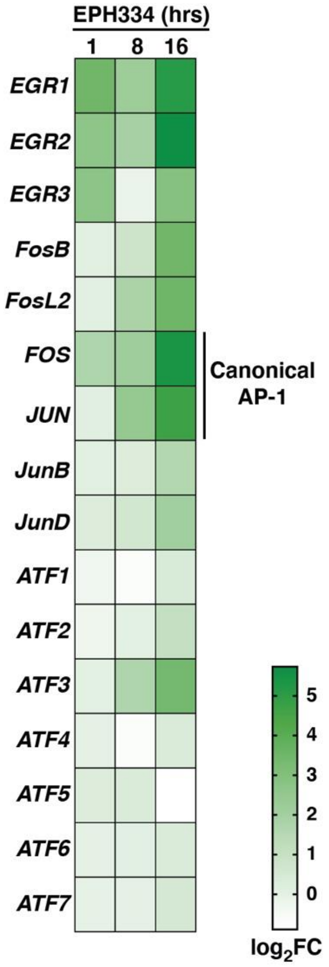

Supplementary Fig. S8. Heatmap of immediate early gene expression in J-Lat 10.6 cell model of latency in response to a temporal EPH334 treatment

log_2FC expression of the indicated genes in J-Lat 10.6 cells treated with EPH334 (2.5 μM) for the indicated time points from the RNA-seq dataset of Fig. 3. The expression of *FOS* and *JUN* encoding the canonical AP-1 subunits (c-Fos and c-Jun, respectively) is indicated.

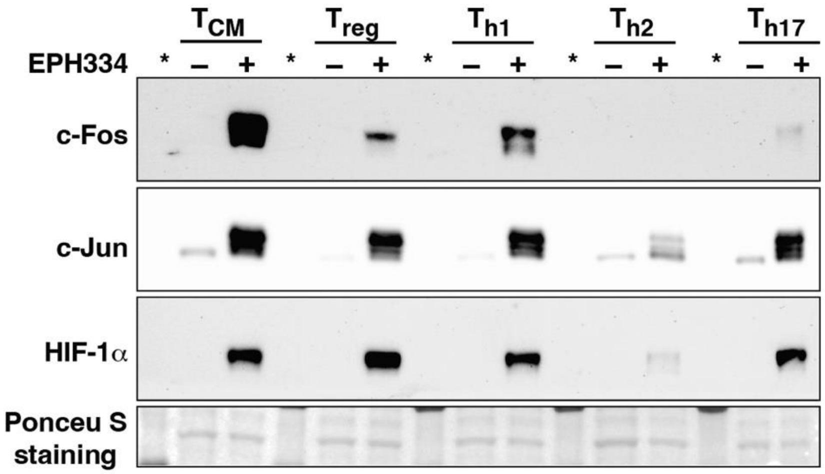

Supplementary Fig. S9. EPH334 induces the redox-responsive master regulators in primary resting CD4$^+$ T cells

Western blots of primary resting CD4$^+$ T cell subsets (T_{CM}, Treg, Th1, Th2, and Th17) showing EPH334 (2.5 μM) treatment for 2 hrs induces c-Fos, c-Jun, and HIF-1α. DMSO (-) was used as vehicle control. Total protein loaded on the gels was revealed by Ponceau S staining. The asterisks (*) denote the presence of a molecular weight ladder separating the indicated lines.

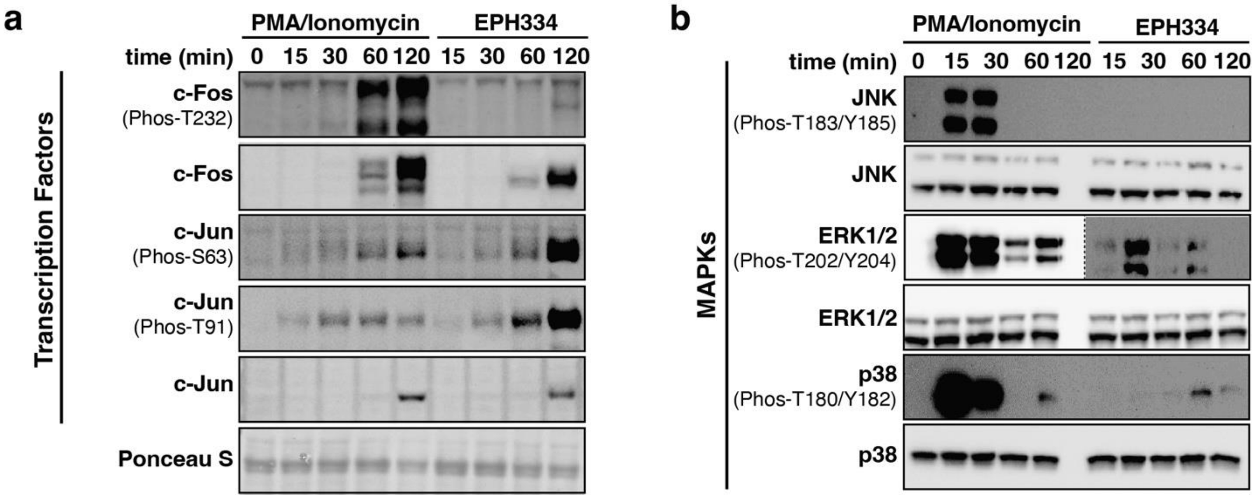

Supplementary Fig. S10. Comparison of PMA/Ionomycin and EPH334 induced AP-1 induction and phosphorylation, and MAPK activation

a) Western blots of J-Lat 10.6 cells treated with the indicated small molecules (EPH334, 2.5 μM; PMA,10 ng/mL) and probed with the indicated antibodies. Total protein loaded was revealed by Ponceau S staining.

b) Western blots of J-Lat 10.6 cells treated with the indicated small molecules (EPH334, 2.5 μM; PMA, 10 ng/mL) and probed with the indicated antibodies. Total protein loaded was revealed by Ponceau S staining. The dashed line indicates the blot was cropped to allow detection of phosphorylated ERK1/2 in both drug treatments because PMA/Ionomycin is a more potent inducer than EPH334. Total JNK, ERK1/2 and p38 serve as internal loading controls as their levels remain unchanged with the treatments.

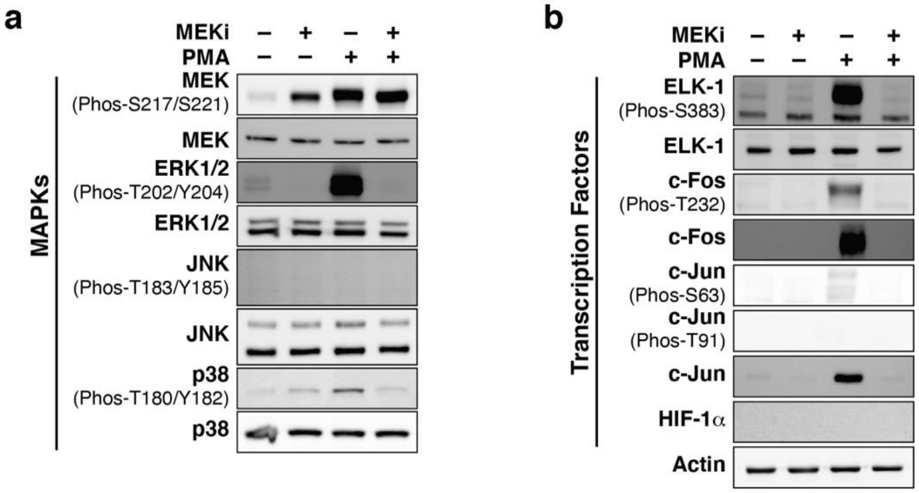

Supplementary Fig. S11. MEK inhibition dampens PMA-induced ERK1/2 phosphorylation, ELK-1 phosphorylation and c-Fos and c-Jun synthesis and phosphorylation

a) Western blots of J-Lat 10.6 cells pre-treated for 1 hr with MEKi (PD0325901, 1 μM) or vehicle control (tissue culture media) and then treated for 15 min with PMA (10 ng/mL) or vehicle control (DMSO) and probed with the indicated antibodies.

b) Western blots of J-Lat 10.6 cells pre-treated for 1 hr with MEKi (PD0325901, 1 μM) or vehicle control (tissue culture media) and then treated for 8 hrs with PMA (10 ng/mL) or vehicle control (DMSO) and probed with the indicated antibodies.

a

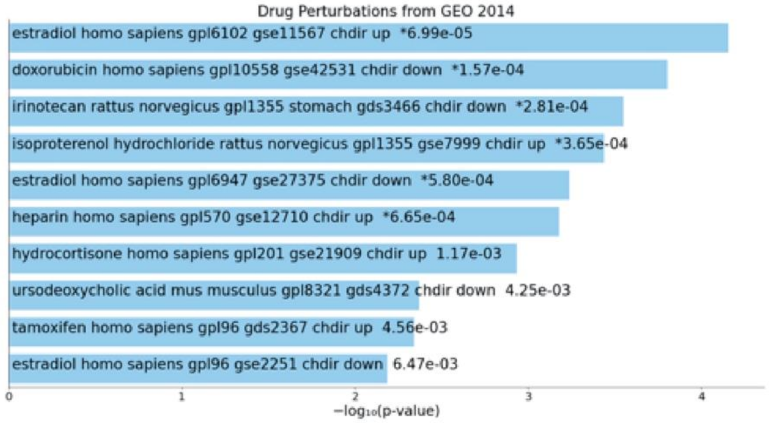

b

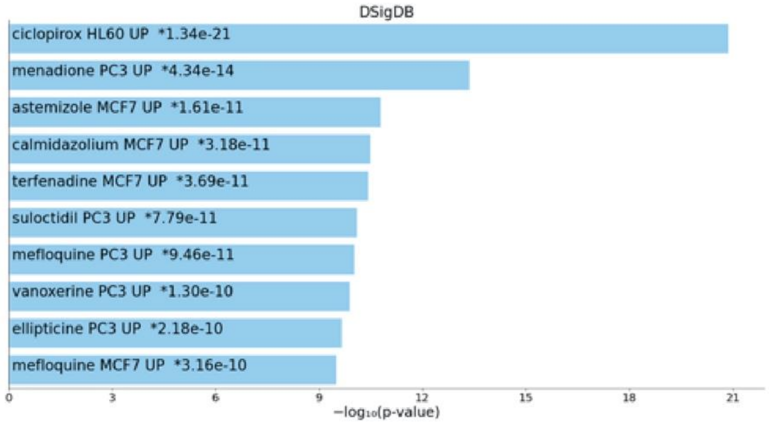

Supplementary Fig. S12. Enrichment of small compounds related to EPH334 through transcriptome profiling analysis with Enrichr

a) Drug perturbations from GEO 2014 enriched in J-Lat 10.6 cells after 1 hr EPH334 (2.5 μM) treatment from Fig. 3 alongside their adjusted p-values.

b) Drug Signatures Database (DSigDB) enriched in J-Lat 10.6 cells after 1 hr EPH334 (2.5 μM) treatment from Fig. 3 alongside their adjusted p-values.

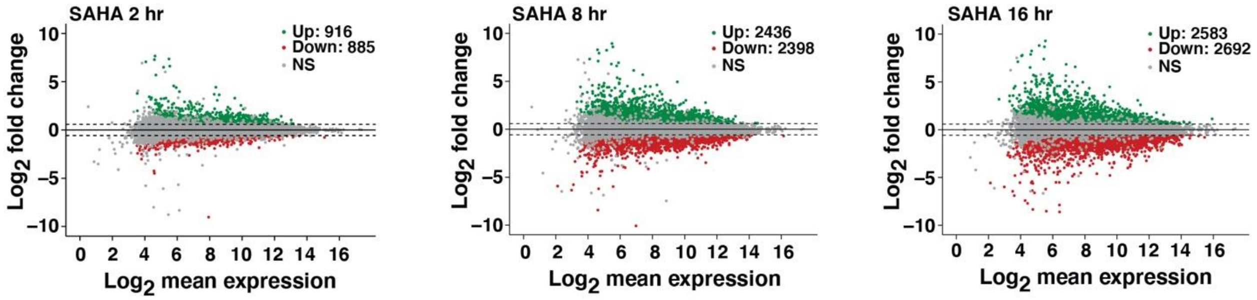

Supplementary Fig. S13. Transcriptional profiling reveals the HDAC inhibitor SAHA induces immediate early genes and a redox-responsive program

Scatterplots of differentially expressed genes identified by RNA-seq of J-Lat 10.6 cells treated with SAHA (1 μM) for the indicated time points (n = 3, false discovery rate [FDR] < 0.05). The number of upregulated (Up), downregulated (Down), and not statistically significant (NS) genes is indicated in each plot. See Supplementary Table 3 for complete list of differentially expressed genes.

Supplemental Tables

Supplementary Table S1. DESeq analysis and list of differentially expressed genes in J-Lat 10.6 cells in response to EPH334 treatment. See attached excel spreadsheet.

Supplementary Table S2. Gene Ontology analysis of differentially expressed genes from Supplementary Table S1. See attached excel spreadsheet.

Supplementary Table S3. DESeq analysis and list of differentially expressed genes in J-Lat 10.6 cells in response to SAHA treatment. See attached excel spreadsheet.

Supplementary Table S4. Cell lines used in this study

**Cell line**	**Laboratory**	**Reference**
J-Lat 10.6	Eric Verdin	Jordan et al., 2003
J-Lat 6.3	Eric Verdin	Jordan et al., 2003
J-Lat 8.4	Eric Verdin	Jordan et al., 2003
J-Lat 9.2	Eric Verdin	Jordan et al., 2003
J-Lat 15.4	Eric Verdin	Jordan et al., 2003
J-Lat E4	Jonathan Karn	Pearson et al., 2008
PBMCs and resting CD4+ T cells	Purified from blood of healthy donors obtained from Gulf Coast Regional Blood Center	Not applicable

Supplementary Table S5. Small compounds used in this study

**Compound**	**Function**	**Source**	**Catalogue number**
TNF-α	NF-κB activator	Sigma	T6674
SCH772984	ERK inhibitor	Selleck Chemical LLC	S7101
PD325901	MEK inhibitor	Selleck Chemical LLC	S1036
(+)-JQ1	Bromo domain inhibitor	APExBio	A1910
SAHA	Pan-HDAC inhibitor	APExBio	A4084
Ionomycin	Calcium ionophore	Sigma	I0634
PMA	PKC agonist	Sigma	P8139
Ingenol 320-dibenzoate	PKC agonist	Enzo Life Sciences	BML-PE186
Menadione	ROS inducer	Sigma	M5625
Raltegravir (ART)	HIV-1 Integrase inhibitor	HIV Reagent Program	ARP-11680
SMORE library	This study		

Supplementary Table S6. siRNAs used in this study

**Target gene**	**Company / Catalogue number**	**Sequence (5'-3')**
Non-targeting (NT)	QIAGEN/ 1027280	Proprietary
Human c-Fos	QIAGEN/ SI02781464	TGGGTTCATTATTGCAATTAA
Human c-Jun	QIAGEN/ SI00300580	AAGAACGTGACAGATGAGCAG
Human HIF-1α	QIAGEN/ SI02664431	AGCCATTTACATAATATAGAA

Supplementary Table S7. DNA oligonucleotides used in this study

Amplicon	Primer Number / Sequence (5'-3')	Assay
HIV-1 US (P1/P2)	1518/TTCT TCAGAGCAGACCAGAGC<br>1519/GCTGCCAAAGAGTGATCTGA	RT-qPCR
HIV-1 SS (P0/P6)	1506/AAAGCTTGCCTTGAGTG CTTCA<br>1508/TATAGGTTGCATTACATGTAC	RT-qPCR
HIV-1MS (P0/P8)	1506/AAAGCTTGCCTTGAGTG CTTCA<br>1507/GATTGGGAGGTGGGTTGCTTTG	RT-qPCR
HIV-1 3'-end (VQA)	1452/CAGATGCTGCATATAAGCAGCTG<br>1453/TTTTTTTTTTTTTTTTTTTTTTTTGAAGCAC	RT-qPCR
HIV-1 Read through	1550/CAGATGCTGCATATAAGCAGCTG<br>1551/CACAACAGACGGGCACACAC	RT-qPCR
HIV-1 +141<br>5'-LTR specific	1111/GCTTAAGCCTCAATAAAGCTTGCCTTGAG<br>1112/GTCCTGCGTCGAGAGATCTCCTCTG	ChIP-qPCR
JUN	856/AGATGAACTCTTTCTGGCCTGCCT<br>857/ACACTGGGCAGGATACCCAAACAA	RT-qPCR
HIF1A	3074/AGCTTGCTCATCAGTTGCCA<br>3075/CCAGAAGTTTCCTCACACGC	RT-qPCR
U6	9/CTCGCTTCGGCAGCACATATAC<br>10/GGAACGCTTCACGAATTTGCGTG	RT-qPCR
RPL19	354/ATCGATCGCCACATGTATCA<br>355/GCGTGCTTCCTTGGTCTTAG	RT-qPCR
FOS	854/ TGACAGATACGCTCCAAGCG<br>855/ TGGCAATCTCGGTCTGCA	RT-qPCR
EGR1	3052/ GGCGAGCAGCCCTACG<br>3053/ GCACCTTCTCGTTGTTCAGAG	RT-qPCR
MT2A	3056/ CCTCCAAGTCCCAGCGAAC<br>3057/ GAGCAGCAGCTTTTCTTGCAG	RT-qPCR
HIST1H2BM	2034/GTACAAGGTGCTGAAGCAGGTC<br>2035/ATCTCCCTCGAAGTGATGGTCG	RT-qPCR
HIST1H2BG	2036/CGATACTGGCATCTCATCCAAGG<br>2037/ATCTCCCTGGAGGTAATGGTCG	RT-qPCR
HIST1H3J	2040/TCCGCAAACTGCCATTTCAGCG<br>2041/GGCGTGAATAGCACAGAGGTTG	RT-qPCR

Supplementary Table S8. Antibodies used in western blot and ChIP assays

Target	Company	Catalogue Number	Assay (Dilution used)
Anti-actin rhodamine	Bio-Rad	12004163	Western blot (1:10000)
β-actin (C-4, mouse monoclonal)	Santa Cruz	sc-47778	Western blot (1:10000)
JNK<br>(2C6, mouse monoclonal)	Cell Signaling	3708	Western blot (1:1000)
T183/Y185 phospho-JNK (G9, mouse monoclonal)	Cell Signaling	9255	Western blot (1:2000)

ERK1/2 (L34F12, mouse monoclonal)	Cell Signaling	4696	Western blot (1:2000)
T202/T204 phospho-ERK1/2 (20G11, rabbit monoclonal)	Cell Signaling	4376	Western blot (1:1000)
p38 (D13E1, rabbit monoclonal)	Cell Signaling	8690	Western blot (1:1000)
T180/Y182 phospho-p38 (3D7, rabbit monoclonal)	Cell Signaling	9215	Western blot (1:1000)
c-Jun (60A8, rabbit monoclonal)	Cell Signaling	9165	Western blot (1:1000)<br>ChIP (2 μg/10x10^6 cells)<br>40 μl = 2 μg
S63 phospho-Jun (mouse monoclonal)	Santa Cruz	sc-822	Western blot (1:2000)
T91 phospho-Jun (Rabbit monoclonal EPR2236)	Abcam	Ab79756	Western blot (1:2000)
Fos (9F6, rabbit monoclonal)	Cell Signaling	2250	Western blot (1:1000)<br>ChIP (2 μg/10x10^6 cells)<br>40 μl = 2 μg
T232 phospho-Fos (rabbit polyclonal)	Abcam	Ab43175	Western blot (1:4000)
Elk-1<br>(Rabbit polyclonal)	Cell Signaling	9182	Western blot (1:1000)
S383 phospho-Elk-1<br>(Rabbit polyclonal)	Cell Signaling	9181	Western blot (1:1000)
HIF-1α<br>Clone tr4 (mouse)	BD Biosciences	610969	Western blot (1:1000)<br>ChIP (2.5 μg/10x10^6 cells)
Normal IgG<br>(Rabbit polyclonal)	Millipore	12-360	ChIP (2 μg/10x10^6 cells)
